# Colloidal InSb Quantum Dots for 1500 nm SWIR Photodetector with Antioxidation of Surface

**DOI:** 10.1002/advs.202306439

**Published:** 2023-11-30

**Authors:** Haewoon Seo, Hyeong Ju Eun, Ah Yeong Lee, Hang Ken Lee, Jong H. Kim, Sang‐Wook Kim

**Affiliations:** ^1^ Department of Molecular Science and Technology AI‐Superconvergence KIURI Translational Research Center Ajou University Suwon Gyeonggi‐do 443–749 Republic of Korea; ^2^ Department of Molecular Science and Technology Ajou University Suwon Gyeonggi‐do 443–749 Republic of Korea; ^3^ Advanced Energy Materials Research Center Korea Research Institute of Chemical Technology (KRICT) Daejeon 34114 Republic of Korea

**Keywords:** III‐V quantum dots, Indium antimony quantum dots, Short wave‐infrared (SWIR) photodetector

## Abstract

III‐V quantum dots (QDs) have emerged as significant alternatives to Cd‐ and Pb‐based QDs, garnering notable attention over the past two decades. However, the understanding of III‐V QDs, particularly in the short wave‐infrared (SWIR) region, remains limited. InAs QDs are widely recognized as the most prominent SWIR QDs, but their absorption beyond 1400 nm presents various challenges. Consequently, InSb QDs with relatively narrower bandgaps have been investigated; however, research on their device applications is lacking. In this study, InSb QDs are synthesized with absorption ranging from 1000 to 1700 nm by introducing Cl^−^ ions to enhance QD surface stability during synthesis. Additionally, it coated InAs and ZnSe shells onto the InSb QDs to validate photoluminescence in the SWIR region and improve photostability. Subsequently, these QDs are employed in the fabrication of photodetector devices, resulting in photodetection above 1500 nm using Pb‐free QDs. The photodetection device exhibited an external quantum efficiency (EQE) of 11.4% at 1370 nm and 6.3% at 1520 nm for InSb core QDs, and 4.6% at 1520 nm for InSb/InAs core/shell QDs, marking the successful implementation of such a device. In detail, the 1520 nm for InSb core device showed a dark current density(J_D_) value of: 1.46 × 10^−9^ A/cm^2^, responsivity(R): 0.078 A/W, and specific detectivity based on the shot noise(D_sh_*): 3.6 × 10^12^ Jones at 0 V.

## Graphical abstract

High‐quality InSb quantum dots(QDs) are synthesized using indium‐carboxylate and tris(trimethylsilyl)antimony(TMS‐Sb) precursors. To mitigate surface oxidation, InCl_3_ is added as a co‐precursor, inducing the presence of Cl^‐^ ions that function as a protective layer on the QD surface. The QD is applied to photo‐detector device, resulting in enhanced EQE values: 11.4% at λ=1370 nm and 6.3% at λ=1520 nm for the InSb core.

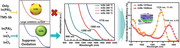

## 1 Introduction

Quantum dots (QDs), nanoscale semiconductor materials with zero‐dimensional characteristics, exhibit distinctive optical properties attributed to the quantum confinement effect resulting from their size. As a result, they have garnered significant attention across various industries and research domains. Since Bawendi et al.’s pioneering work in synthesizing colloidal type CdSe QDs,^[^ ^1^ ^]^ numerous QDs have been successfully synthesized and reported, exhibiting their unique characteristics across a broad spectrum ranging from ultraviolet (UV) to near‐infrared (NIR). Extensive research is being conducted in diverse fields, including LEDs,^[^ ^2^, ^3^, ^4^, ^5^ ^]^ solar energy harvesting,^[^ ^6^, ^7^, ^8^, ^9^ ^]^ photodetectors,^[^ ^10^, ^11^, ^12^, ^13^ ^]^ and bio‐markers.^[^ ^14^, ^15^, ^16^, ^17^ ^]^ Notably, the application of QDs in NIR‐SWIR photodetectors has recently gained significant attention.

The remarkable progress in optical instruments has fueled a substantial upsurge of interest in SWIR sensing. The NIR‐SWIR region stands out for its exceptional capability in object recognition during low‐light or nighttime situations, owing to its reduced autofluorescence and scattering properties when compared to the visible region.^[^ ^18^, ^19^ ^]^ As a result, materials with absorption in the NIR‐SWIR region have gained significant attention within the field of photodetectors. To date, Pb chalcogenide (PbX(S, Se, Te)) QDs have emerged as the most extensively studied QDs exhibiting absorption in the NIR‐SWIR region. These QDs belong to the typical IV‐VI group and offer exceptional ease of synthesis and control over particle size. Pb chalcogenide QDs exhibit remarkable optical properties and stability. By adjusting the combination of chalcogen elements (S, Se, and Te), the wavelength can be finely tuned from 800 to 2400 nm. As a result, Pb chalcogenide QDs have found widespread applications in NIR solar cells^[^ ^20^, ^21^, ^22^ ^]^ and photodetectors^[^ ^23^, ^24^, ^25^ ^]^ thus far. Nevertheless, it is well‐known that Pb, along with Cd and Hg, fall under the category of high toxic heavy metals regulated by RoHS.^[^ ^26^ ^]^ Consequently, research efforts are actively underway to develop Pb‐free QDs for the NIR region, taking environmental considerations into account. Several Pb‐free QD alternatives for the NIR region have been introduced, including InAs,^[^ ^27^, ^28^ ^]^ CuInSe,^[^ ^29^, ^30^ ^]^ and Ag_2_S;^[^ ^31^, ^32^ ^]^ among them, III‐V semiconductor InAs QDs have attracted the most extensive research. InAs QDs have made significant advancements in performance, drawing upon the wealth of studies on InP QDs. InP, being a member of the same III‐V QD family, has undergone numerous research and optimization processes to match the quality of CdSe‐based QDs in the visible light region. Building upon this progress, InAs QDs have rapidly enhanced their performance as well. Recently, S. Jeong et al. successfully achieved the synthesis of InAs QDs with uniform size distribution^[^ ^33^ ^]^ and excellent optical properties^[^ ^34^ ^]^ in the 1400–1600 nm region through optimized synthesis methods and surface treatment. Additionally, E. Sargent et al. accomplished an impressive EQE performance exceeding 30% at 920 nm by applying InBr treatment to the surface of InAs QDs.^[^ ^35^ ^]^ However, in order to extend the absorption range of InAs QDs to over 1500 nm, the particle size must be at least 10 nm. Achieving uniform growth to such a size requires meticulous control and extensive optimization during the synthesis process. Considering this perspective, InSb QDs have garnered attention as Pb‐free QDs due to their absorption capabilities in the deep NIR region, despite their relatively smaller size.

InSb, a III‐V QD, possesses a narrower bandgap of 0.17 eV compared to InAs with 0.354 eV. D. Talapin et al. successfully synthesized InSb cores for the first time using InCl_3_, Sb[N(Si(Me)_3_)_2_]_3_ precursors, and LiEt_3_BH as a reducing agent. These InSb QDs exhibit a distinctive absorption shoulder peak above 1500 nm.^[^ ^36^ ^]^ Additionally, Kagan et al. synthesized InSb_x_As_(1‐x)_ QDs using the same method as mentioned earlier, demonstrating the ability to adjust the absorption region from 900 to 1800 nm by controlling the Sb and As precursor ratio. They also showcased the performance of NIR photodetectors with field effect transistor (FET)‐based devices.^[^ ^37^ ^]^ However, this synthesis method involving reducing agents often results in poorer size distribution compared to conventional methods, necessitating a size selection process during synthesis. Consequently, we attempted a different approach using InCl_3_‐carboxylate and tris(trimethylsilyl)pnictogen (TMS‐pnictogen) precursors, which are known as representative methods for synthesizing III‐V QDs. The carboxylate‐TMS system offers the advantage of a simplified synthesis process that can utilize both hot injection and heat‐up processes. In our study, we synthesized InSb QDs by injecting the Sb precursor at a low temperature of 75°C and subsequently raising the temperature to promote QD growth. By controlling the growth temperature, we achieved particle sizes ranging from 2 to 6 nm and confirmed an absorption peak range of 1000 to 1700 nm for the QDs. Remarkably, the utilization of chloride ions for robust surface protection demonstrated an intriguing outcome—the prevention of Sb oxidation, consequently leading to enhanced absorption characteristics in InSb QDs. We further synthesized a type‐I InSb/InAs core/shell QD by coating the surface with an InAs shell, observing enhanced photoluminescence (PL) in the SWIR region, which was previously unattainable in the core structure. Furthermore, we introduced a ZnSe II‐VI outer shell to complete the core/shell/shell structure, thereby increasing optical stability and enhancing the PL spectra. Building upon these findings, we fabricated photodiode‐type photodetector devices using InSb QDs absorbing 1370 nm, 1520 nm region, and InSb/InAs QDs absorbing 1520 nm, achieving EQEs of 11.4%, 6.3%, and 4.6% respectively.

## 2 Results and Discussion

**Figure 1:**
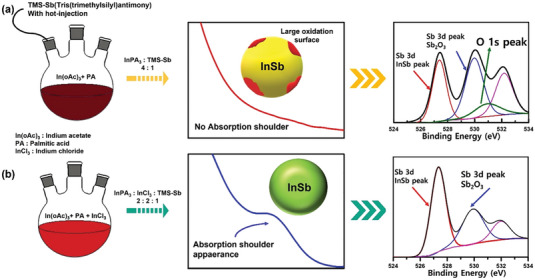
InSb QD properties depending on precursors (schematic illustration, absorption spectrum, XPS peak fitting result) a) synthesis without InCl_3_ b) synthesis with InCl_3._

As previously mentioned, we synthesized InSb core QDs using indium‐carboxylate and tris(trimethylsilyl)antimony (TMS‐Sb) as indium and antimony precursors, respectively. However, no shoulder peak was observed in the absorption spectrum of InSb cores, despite various experimental conditions.^[^ ^38^, ^39^ ^]^ To address this issue, the focus was directed towards the surface of the QDs. Based on previous reports,^[^ ^40^ ^]^ it was determined that the subpar optical properties of QDs primarily stem from surface‐related issues. The optical properties of QDs, particularly III‐V QDs, are significantly impaired compared to II‐VI and II‐IV QDs when the QD surface undergoes oxidation or contains dangling bonds.^[^ ^41^ ^]^ To address this issue, many research groups have attempted surface halide passivation or the incorporation of III‐V/II‐VI core/shell structures as solutions to improve the surface properties of III‐V QDs.^[^ ^42^, ^43^, ^44^ ^]^ Kwon et al. successfully synthesized high‐quality InP cores using an InCl_3_‐carboxylate precursor, achieving exceptional quality due to the etching and surface passivation effects of Cl^−^ ions.^[^ ^45^ ^]^ Based on this result, we employed the mixture precursors of InCl_3_‐TOP(tri‐n‐octylphosphine) and In‐carboxylate, resulting in successful halide surface passivation during InSb core synthesis. This approach led to the production of InSb cores with stable optical properties. From the schematic illustration of synthesis (**Figure** **1**, left), the mixtures of InPA_3_ and InCl_3_ complex solution exhibit a significantly brighter red color compared to the batch containing only InPA_3_ immediately after the injection of TMS‐Sb. Despite the growth process, the absorption spectrum of InSb QDs with InPA_3_ alone does not exhibit an absorption peak shoulder. Conversely, when the synthesis is carried out in conjunction with the InCl_3_ complex, an absorption shoulder becomes apparent. This phenomenon can be attributed to the inhibitory effect of Cl^−^ on the surface of the InSb core, which helps suppress oxidation.^[^ ^43^, ^46^, ^47^, ^48^ ^]^ The X‐ray photoelectron spectroscopy (XPS) measurements of each core reveal that only the InPA_3_ sample exhibits the presence of an O 1s peak in the Sb 3d XPS spectrum. The Sb_2_O_3_ peak is believed to be a result of the decarboxylation process from palmitic acid during synthesis. The Sb_2_O_3_ peak at 530 eV in the InPA_3_ sample appears larger in size compared to the peak observed in the sample with a mixture of InPA_3_ and InCl_3_. This suggests that the presence of Cl^−^ suppresses surface oxidation, leading to improved optical properties. By comparing the 3d peak positions of Sb, the peak of the InCl_3_ sample was shifted to a slightly higher energy than that of the InPA_3_ sample (Figure S1, Supporting Information), owing to the attachment of Cl^−^ to the QDs surface, as Cl^−^ is a stronger X‐type ligand than PA. This observation was further supported by the TGA analysis of the two samples, revealing that the final residue of the sample with InCl_3_ was 70% in the TGA plot. This confirmed that the proportion of volatile organics in the sample with InCl_3_ was lower than the 58.6% residue observed in the InPA_3_ sample (Figure S2, Supporting Information). It was thus inferred that the presence of Cl^−^ effectively suppressed the surface oxidation of the QD surface.

**Figure 2:**
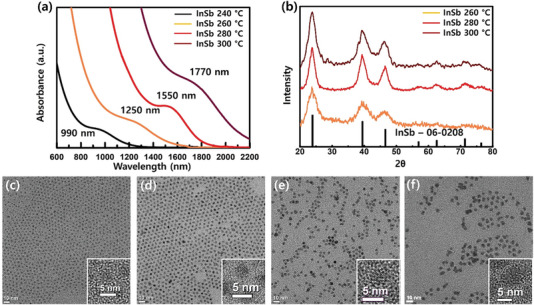
a) Absorbance spectra of InSb QDs depending on growth temperature (black: 240 °C, yellow brown : 260 °C, red: 280 °C, dark brown : 300°C). (b) XRD spectra dependent on growth temperature (yellow brown : 260 °C, red: 280 °C, dark brown : 300 °C). TEM image of InSb QDs c) 240°C growth, d) 260°C growth, e) 280°C growth, f) 300 °C growth_._

Cores of various sizes were synthesized based on a mixture of InCl_3_‐TOP and In‐carboxylate precursors by controlling the growth temperature between 240–300 °C for 10 min. According to the absorption spectrum in **Figure** **2**a, the following absorption peaks were observed: 990 nm at a growth temperature of 240 °C, 1250 nm at 260 °C, 1550 nm at 280 °C, and 1770 nm at 300 °C. All cores exhibit X‐ray diffraction (XRD) spectra indicating the InSb zinc‐blende crystal structure, as shown in Figure 2b. Depending on the growth temperature, the cores synthesized at 240 and 260 °C have a size of ≈2 and 3 nm, respectively, as depicted in Figure 2c,d. Thereafter, the cores synthesized at 280 °C reach a size ranging from 4 to 5 nm, as illustrated in Figure 2e. At growth temperatures exceeding 280 °C, the size distribution becomes unfavorable, and there is a tendency for particles to agglomerate with each other (Figure 2f). We additionally calculated the size distribution for each particle, and the size distribution appeared relatively uniform (Figure S3, Supporting Information). Each synthesis reaction was conducted with an In to Sb ratio of 4:1. However, when the portion of Sb was increased with In:Sb ratios of 3:1 and 2:1, the absorption peak broadened and redshifted, as shown in Figure S4a, Supporting Information. On the other hand, when the portion of Sb was decreased to 6:1, there was no significant difference compared to the 4:1 ratios, except for a slight broadening as depicted in Figure S4b, Supporting Information. In these experiments, the ratio of InPA_3_ to InCl_3_ was kept at 1:1. As the proportion of InCl_3_ increased, there was a relative blue shift in the peak position, and the peak shoulder exhibited widening, as shown in Figure S5a, Supporting Information. This can be attributed to the increased presence of Cl^−^ ligands, which are known to strongly attach to the surface of QDs, leading to poor size distribution. On the contrary, when the proportion of InPA_3_ increased, the shape of the peak became unclear. This phenomenon can be attributed to the higher amounts of PA, which increase decarboxylation and further accelerate surface oxidation, as depicted in Figure S5b, Supporting Information.

Subsequently, we applied the coating of an InAs shell onto the surface of the InSb core. At this stage, the disparity in crystal lattice parameters became a primary consideration in the selection of the shell material. A significant difference in lattice parameters between the core and the shell not only hinders the successful epitaxial coating of the shell on the surface, but also creates additional trap sites at the core/shell interface. These trap sites pose a significant challenge to the overall properties of the QDs.

**Figure 3:**
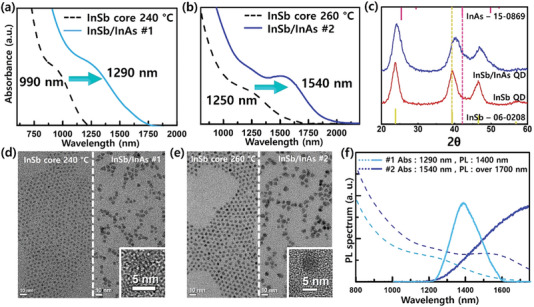
Characterization of InSb core and InSb/InAs core/shell QDs. a) Absorption of InSb 240 °C growth core (black dash line) and InSb/InAs core/shell #1 QDs (azure line). b) Absorption of InSb 260 °C growth core (black dash line) and InSb/InAs core/shell #2 QDs (blue line). c) XRD XRD spectrum of InSb core and InSb/InAs core/shell QDs. TEM image of d) 240 °C growth core and InSb/InAs core/shell #1 QDs, e) 260 °C growth core and InSb/InAs core/shell #2 QDs. f) Absorption (azure & blue dash line) and PL spectra (azure, blue line) of InSb/InAs core/shell QDs.

The lattice parameter of InSb is 6.47 Å, and suitable II‐VI semiconductor materials for the shell include CdS (5.84 Å), CdSe (6.05 Å), CdTe (6.48 Å), and ZnTe (6.18 Å). Previous reports by Tarapin et al.^[^ ^36^ ^]^ have demonstrated successful shell coating using these materials. However, ZnSe (5.66 Å) and ZnS (5.42 Å) are not suitable for coating on InSb surfaces due to their large lattice mismatch. Considering this, we opted for the III‐V semiconductor InAs as the shell material on the InSb surface. InAs, like InSb, has a zinc‐blende structure with a crystal lattice parameter of 0.605 nm, which is similar to InSb's crystal lattice parameter of 0.648 nm. This similarity makes InAs an appropriate choice for achieving a type‐I core/shell structure. We conducted the InAs shell coating using the hot injection method at a temperature of 280°C. For the InAs complex as a shell precursor, we mixed InCl_3_ and TMS‐As in TOP at a ratio of In:As = 3:2. During the injection step, the InAs complex was divided and sequentially injected at 5 min intervals, repeating the process for a total of n times (*n* = 4 to 8, 0.25 mmol/injection) injections. The InAs shell coating was performed using the mentioned InAs complex on the InSb core of 1340 nm absorption peak. As a result, depending on the amount of InAs complex implanted, the InSb/InAs QDs exhibited absorption peaks at 1518 nm (*n* = 4, total InAs complex amount = 1 mmol), 1590 nm (*n* = 6, InAs 1.5 mmol), and 1750 nm (*n* = 8, InAs 2 mmol) (Figure S6, Supporting Information). We aimed to achieve core/shell QDs with a thick shell for improved stability and an absorption peak at 1500 nm. However, when using InSb cores with an absorption peak exceeding 1300 nm, even a small amount of InAs complex led to an extension of the absorption peak beyond the desired 1500 nm region, resulting in peak broadening. To address this, we performed the shell coating on smaller InSb cores to successfully synthesize core/shell QDs with a distinct absorption shoulder in the 1500 nm region. **Figure** **3** shows the InSb/InAs core/shell QDs with shell coating on two smaller‐sized InSb cores. Figure 3a,b are the absorption spectra of InSb/InAs core/shell QDs, which showed the red‐shifted absorption from 990 to 1290 nm and from 1250 to 1540 nm, respectively. After shell coating, a significant redshift of 300 nm in the absorption peak was observed. This substantial redshift is associated with the narrow bandgap of InAs. Typically, even in type‐I core/shell QDs, a red‐shift in the absorption peak is commonly observed. This phenomenon is interpreted as resulting from partial leakage into the shell of the radial probability wave functions of electron and hole. From this perspective, a narrower bandgap in the shell leads to more pronounced wave function leakage, causing a more distinct red‐shift phenomenon.^[^ ^40^, ^49^ ^]^ In our work, InSb/InAs QDs exhibit a significant redshift due to the notably narrow bandgap of InAs (InAs: 0.35 eV) comparing with CdSe (1.74 eV) or CdS (2.42 eV). Furthermore, we observed small redshift when we reduced the InAs complex during the shell‐coating process, as elucidated in Figure 3b. (Figure S7, Supporting Information) From the XRD spectrum shown in Figure 3c, it can be observed that each peak shifted towards the zinc‐blende InAs reference while maintaining the zinc‐blende crystal structure after the coating of the InAs shell. This shift in peak positions indicates the successful completion of the core/shell structure. Figure 3d,e displays transmission electron microscopy (TEM) images of two different‐sized InSb cores and their corresponding InSb/InAs QDs, respectively. In both cases, an increase in size of approximately 2 nm or more (Figure 3d: 2–3 to 4–5 nm, Figure 3e: 3–4 to 5–6 nm) is observed compared to the core size. These results confirm that the InAs shell is effectively coated on the surface of the InSb core. Figure 3f shows the absorption and PL spectra of two samples. The InSb core did not exhibit any PL characteristics, despite its strong absorption. However, the InSb/InAs QDs demonstrated luminescence, which can be attributed to their type‐I structural features. Moreover, the core/shell QDs also exhibited enhanced photostability. Figure S9, Supporting Information illustrates the change in the absorption spectrum over time for both the InSb core and the InSb/InAs core/shell QDs. In the case of the InSb core, the shape of the absorption shoulder significantly diminished after 36 h, whereas the InSb/InAs core/shell QDs maintained the absorption shoulder even after 84 h. This suggests that the InAs shell had a positive impact on improving the overall optical performance of the InSb core. We measured time‐correlated single photon counting (TCSPC) to measure fluorescence decay in the time domain. The single‐exponential decay analysis showed that the τ1 value was 84.6 ns for the InSb core and 52.6 ns for the InSb/InAs core/shell, indicating a lower τ1 value for the core/shell sample. In single‐exponential decay, a fast decay value of τ1 corresponds to exciton radiative recombination, and this result also suggests that the core/shell is a type‐I structure (Figure S8, Supporting Information).

Building upon the results of the InSb/InAs core/shell QDs, we proceeded to coat a ZnSe outer shell onto the InAs shell surface. It is commonly observed that II‐VI shell coatings are utilized to compensate for surface defects in III‐V QDs, leading to improved optical performance and stability. Numerous studies have reported the successful application of II‐VI shells onto InP and InAs QD cores.^[^ ^27^, ^42^, ^50^, ^51^ ^]^

**Figure 4:**
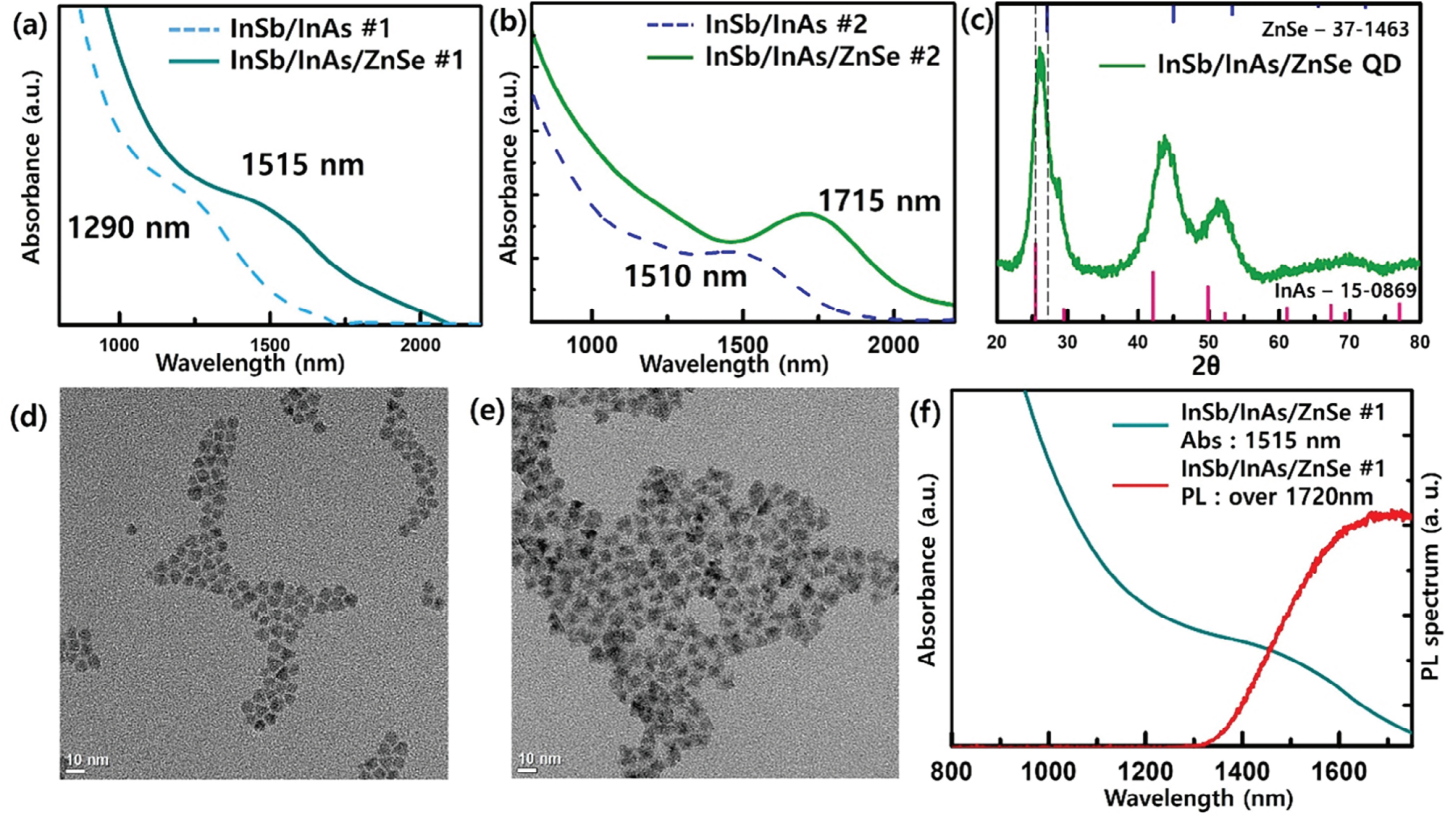
Characterization of InSb core and InSb/InAs core/shell QDs. a) Absorption of InSb 240 °C growth core (azure dash line) and InSb/InAs core/shell #1 QDs (cyan line). b) Absorption of InSb 260 °C growth core (blue line) and InSb/InAs core/shell #2 QDs (green line). c) XRD XRD spectrum of InSb core and InSb/InAs core/shell QDs. TEM image of d) 240 °C growth core and InSb/InAs core/shell #1 QDs, e) 260°C growth core and InSb/InAs core/shell #2 QDs. f) Absorption (cyan line) and PL spectra (red line) of InSb/InAs core/shell QDs.

As mentioned earlier, the key factor in successfully coating a III‐V core with an II‐VI shell is reducing the lattice mismatch between the core and the shell. Therefore, we introduced a ZnSe (5.66 Å) outer shell onto the InSb/InAs (6.05 Å) QDs, aiming to synthesize QDs with a core/shell/shell structure. **Figure** **4**a,b present the absorption spectra of the InSb/InAs/ZnSe core/shell/shell QDs. Upon shell coating, the absorption shoulder peak underwent a redshift of approximately 200 nm, similar to the behavior observed with the InAs shell. However, the shape of the peak was well‐maintained. Consistent with the InAs shell case, the XRD spectrum also showed a peak shift towards the ZnSe reference, as shown in Figure 4c. TEM analysis confirmed a significant increase in particle size (Figure 4d,e). Furthermore, energy dispersive spectroscopy (EDS) analysis confirmed that the elements In, Sb, Zn, and Se were detected at the same location, indicating their presence within the same region of the QDs (Figure S10, Supporting Information).

In this core/shell/shell structure, each shell maintains a type‐I structure with the InSb core, enabling PL to be observed in the core/shell/shell QDs, with an absorption peak at 1515 nm. Furthermore, the II‐VI coating led to an increase in photostability, as shown in Figure 4f. For the core/shell/shell QDs, the absorption peak shape was maintained for approximately 196 h, significantly longer than that observed for the InSb/InAs core/shell QDs (Figure S11, Supporting Information).

We successfully fabricated a photodiode‐type photodetector (PD) device using previously synthesized InSb and InSb/InAs QDs and examined its effectiveness as a device. As mentioned earlier, it is essential to mitigate the interference from moisture in the SWIR region. Considering this aspect, QDs with specific absorption characteristics outside the water absorption bands were synthesized and incorporated into the photodetector device fabrication process. Particularly, QDs exhibiting absorption at 1370 and 1520 nm were used for the photodetector, effectively avoiding the influence of water absorption. This allowed for a precise performance comparison between the two types of QDs within a SWIR wavelength range, enabling the most reliable measurement of EQE.

**Figure 5:**
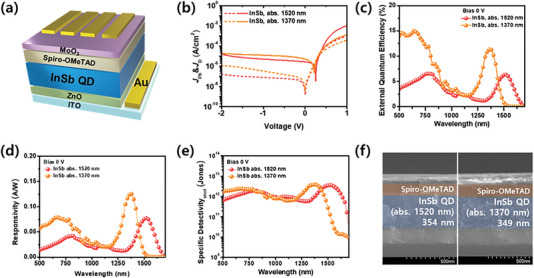
a) Schematic diagram of InSb photodetector device. b) *J*−*V* characteristic in dark and under SWIR light (wavelength: 1370, 1520 nm). Device performance of InSb‐based device under zero bias c) EQE, d) responsivity, e) specific detectivity based on the shot noise. f) Side view of SEM image of photodetector device.

The architecture of the fabricated device is illustrated in the schematic depicted in **Figure** **5**a. The device structure is ITO/ZnO/QD/Spiro‐OMeTAD/MoO_3_/Au. The highest occupied molecular orbital (HOMO) levels of the InSb and InSb/InAs QDs were measured using ultraviolet photoelectron spectroscopy (UPS). The HOMO levels of the QD active layer were determined to be 5.41 eV for InSb and 5.5 eV for InSb/InAs. These measurements were utilized to construct the band diagram of the entire device (Figure S12, S13, Supporting Information). In the case of the III‐V QDs, surface oxidation significantly impedes overall optical performance, especially when the anionic sites of the pnictogen series oxidize, leading to the formation of deep traps within the bandgap which can be detrimental.^[^ ^52^ ^]^ The presence of these traps tends to increase the recombination of photocurrent and the level of dark current in the performance of devices, which can severely degrade the device's performance. To overcome this, we fabricated the device using a cationic molecular metal chalcogenide (MCC), which can effectively protect the Sb sites, as opposed to ligands like EDT or halide. Here, we introduced the cationic molecular metal Mn_2_Se_2_ as the cationic MCC ligand and spin‐coated the QD thin film for device fabrication.^[^ ^53^ ^]^ We focused on protecting the Sb portion of the QDs using this cationic MCC ligand. This MCC ligand prevents potential oxidation during the device fabrication process, eliminating surface traps, and suppressing the dark current and the recombination of photocurrent.

Upon cationic MCC ligand exchange, the first Abs. peak of the QD solution was red‐shifted from 1490 to 1515 nm for the InSb core, and from 1500 to 1515 nm for the InSb/InAs core/shell, respectively (Figure S14, Supporting Information). When the XPS analysis of the MCC‐InSb core was conducted, the peak associated with InSb exhibited a slight redshift towards higher energy. This phenomenon can be attributed to the binding of the cationic MCC ligand to the Sb atoms, consistent with previously reported results^[^ ^53^ ^]^ (Figure S15, Supporting Information).

The figure of merits in the performance of a photodiode‐type photodetector (PD) device is the specific detectivity which can be estimated based on shot noise (D_sh_*) and noise spectral density (D_n_*). We obtained spectral D_sh_* and D_n_* characterized (Figure 5b) using the following equations.^[^ ^54^ ^]^

(1) $$D_{\mathrm{sh}}^{*}=\frac{R\sqrt{A}}{\sqrt{2qI_{D}}}=\frac{R}{\sqrt{2qJ_{D}}}$$

(2) $$D_{n}^{*}=\frac{R\sqrt{A}}{S_{n}}$$

where R denotes the responsivity, A is the device area, q is the electrical charge, I_D_ denotes the dark current intensity, J_D_ is the dark current density, and S_n_ is the noise spectral density. The InSb core device exhibited a value of J_D_: 3.3×10^−9^ A/cm^2^, R: 0.126 A/W, D_sh_*: 3.9 × 10^12^ Jones at 0 V in the 1370 nm region, and J_D_: 1.5×10^−9^ A/cm^2^, R : 0.078 A/W, D_sh_* : 3.6 × 10^12^ Jones at the 1520 nm region. To obtain D_n_*, we extracted noise spectral density for two devices. As shown in Figure 5b, both the 1370 nm device and the 1520 nm device exhibited 1/f behavior while the 1370 nm device showed saturation at frequency over 10 Hz. Figure S18, Supporting Information presents spectral D_n_* for the InSb core devices, in which D_n_* for 1370 and 1520 nm were obtained to be 1.3 × 10^12^ and 1.3 × 10^11^ Jones, respectively. As presented in Figure 5f, the thickness of the QD photoactive layer was 349 nm in the 1370 nm device and 354 nm in the 1520 nm device. During the device fabrication process, we conducted experiments to determine the optimal thickness of the photoactive layer. Through these experiments, we discovered that thicknesses ranging from 350 to 400 nm yielded the most favorable results. For comparison, we also measured the absorbance of QD films with thicknesses of 177, 354, and 579 nm. Among these samples, the 354 nm QD film exhibited the highest absorption coefficient in the absorption shoulder region (Figure S16, Supporting Information). This is somewhat consistent with the results reported by Sargent et al.^[^ ^55^, ^56^ ^]^ Previous reports indicated that higher efficiencies can be achieved with thicker active layers. However, in practical applications, achieving thicknesses above 500 nm without defects can be extremely challenging. Therefore, for the devices we fabricated, we opted for a QD photoactive layer thickness ranging from 350 to 400 nm. This range allowed us to strike a balance between maximizing efficiency and avoiding the complications associated with excessively thick layers. In particular, we confirmed remarkable EQE performances of 11.4% and 6.3% in the SWIR region of 1370 and 1520 nm, respectively. To investigate the reliability of the devices, we measured the linear dynamic range (LDR) of the InSb core devices. As shown in Figure S19, Supporting Information, LDR for the 1370 and 1520 nm devices are evaluated to be 57 and 68 dB, respectively, wherein higher dynamic range was achieved for the 1520 nm device owing to lower dark current density. In addition, the power density of illuminated light used for EQE measurements was measured to be 25 at 1370 nm and 32 mW/cm^2^ at 1520 nm. These power densities are included in light intensity regime in LDR characterization, supporting the reliability of EQE measurements. These results show the highest performance among InSb series QDs reported to date (Table S1, Supporting Information). This confirmed that the antioxidation effect of the InSb surface was effective.

**Figure 6:**
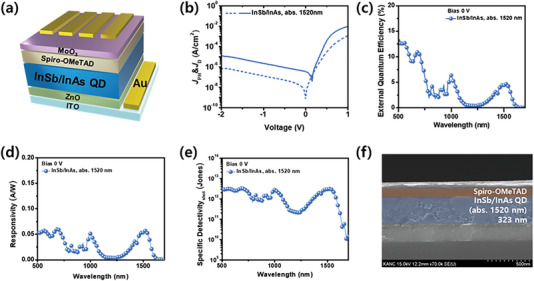
a) Schematic diagram of InSb/InAs photodetector device. b) *J*−*V* characteristic in dark and under SWIR light (wavelength: 1520 nm). Device performance of InSb/InAs based device under zero bias c) EQE, d) responsivity, e) specific detectivity based on the shot noise. f) Side view of SEM image of photodetector device.

Based on the above results, a device using InSb/InAs core/shell QDs was fabricated with the same structure as mentioned earlier (**Figure** **6**). The device exhibited a performance of J_D_: 1.07×10^−9^ A/cm^2^, EQE: 4.64%, R: 0.057 A/W, and D_sh_*: 3.07 × 10^12^ Jones at 0 V. Furthermore, the thickness of the active layer in the device was set at a similar value of 323 nm, comparable to that of the core devices (Figure 6e). The core/shell device showed a slightly lower result than the core. This difference can be attributed to the fact that the InSb/InAs core/shell QDs have a type‐I structure, as illustrated in Figure 3. In this structure, the performance of PD devices tends to be slightly compromised due to increased exciton recombination in the QDs, as previously mentioned. This phenomenon leads to slightly inferior performance compared to devices utilizing only core QDs. Nevertheless, these results indicate that QDs with a type‐I core cell structure can also form EQEs successfully in the SWIR region.

## 3 Conclusion

High‐quality InSb QDs were synthesized using indium‐carboxylate and TMS‐Sb precursors. To mitigate surface oxidation of the QDs, we introduced InCl_3_ as a co‐precursor, inducing the presence of Cl^−^ ions that functioned as a protective layer on the QD surface.

Our InSb QD synthesis process distinguishes itself from conventional methods by not requiring reducing agents for the In and Sb precursors. This approach offers a rapid and straightforward process, resulting in a relatively uniform size distribution of the QDs. These notable advantages render our method highly efficient and convenient for InSb QD production. By encapsulating the core InSb QDs with InAs and ZnSe shells, we observed photoluminescence in the SWIR region, signifying a significant enhancement in photostability. The core/shell QDs exhibited type‐I properties, characterized by red‐shifted absorption and faster decay times.

Subsequently, we applied the InSb core and InSb/InAs core/shell QDs to PD devices. To minimize dark current in the devices, we employed cationic MCC ligands, resulting in enhanced EQE values: 11.4% at λ = 1370 nm and 6.3% at λ = 1520 nm for the InSb core, and 4.6% at λ = 1520 nm for the InSb/InAs core/shell in the SWIR absorption region.

We successfully synthesized QDs with sharp absorption peaks in the SWIR region using a synthesis method involving Cl^−^ ions. Our approach yielded improved optical performance, underscoring the efficacy of a simplified synthesis protocol that eliminates the need for reducing agents or size‐selection processes (see Table S1, Supporting Information). We fabricated photodiode‐based photodetector devices and achieved EQE values surpassing the 1500 nm threshold for the first time, demonstrating markedly superior device performance compared to other reported Pb‐free QDs (see Figure S17, Supporting Information). These results point to the promising potential of InSb QDs as lead‐free alternatives with favorable optical properties in the SWIR region. This discovery holds substantial implications for expanding the application scope of QDs in various devices.

## 4 Experimental Section

### Materials

All chemicals, indium acetate (In(OAc)_3_ 99.99% trace metal basis, Sigma–Aldrich), indium chloride (InCl_3_, 99,99%, Sigma–Aldrich), zinc acetate (Zn(OAc)_2_ 99.99% trace metal base, Sigma–Aldrich), palmitic acid (99%, Sigma), oleic acid (90% technical grade, Aldrich), selenium (Se, pellets, 99.999%, Sigma‐Aldrich), hexane (anhydrous, Sigma‐Aldrich), tetrachloroethylene (TCE, anhydrous, Sigma–Aldrich), tri‐n‐octylphosphine (TOP, 97%, Strem), tris(trimethylsilyl)arsine (TMS‐As, 99%, JSI‐silicon), tris(trimethylsilyl)antimony (TMS‐Sb, 99%, JSI‐silicon) were used without any further purification.

### Synthesis of InSb core QDs

For the TOP‐InCl_3_ solution, InCl_3_ (0.442 g, 2 mmol) was dissolved in TOP of 8 ml with vigorous stirring at room temperature (RT, 25°C) for 1 day. For the synthesis of InSb QDs, indium acetate (0.584 g, 2 mmol), palmitic acid (1.538 g, 6 mmol) was dissolved in TOP of 12 ml. The resulting solution was then degassed for 10 min at RT. After degassing, In precursor solution was heated to 150 °C under the N_2_ atmosphere using an outgassing flow system. After 1 hour, 8 ml TOP‐InCl_3_ complex solution was added to In(PA)_3_ solution and maintain temperature for 1 h. Subsequently, 0.341 mg of TMS‐Sb (1 mmol) in 1 ml hexane was injected into the In precursor solution at 75 °C and raise temperature for 3 °C/min to target temperature (240–300 °C). Growth time is 10 min. The product was obtained by the centrifuge (8000 rpm, 10 min) with acetone and hexane.

### Synthesis of InSb/InAs Core/Shell QDs

For the InAs shell coating, InAs complex solution was first prepared. It was prepared by mixing the TOP‐InCl_3_ solution and TMS‐As precursor at RT in a 3:2 ratio of In:As. The appropriate amount of InAs solution was injected into InSb core solution at 280 °C and maintained for 10 min. The product was obtained by the centrifuge (8000 rpm, 10 min) with acetone and hexane.

### For InSb/InAs/ZnSe Core/Shell/Shell

Zinc acetate (732 mg, 4 mmol) and oleic acid (2.8 g, 1 mmol) were dissolved in 50 ml ODE, then, the solution was degassed for 10 min at RT. The solution temperature was raised to 280 °C under the N_2_ atmosphere with outgassing. Then, InSb/InAs QD solution was injected into the solution, followed by the immediate addition of 1.4 ml of a 10% diluted hydrofluoric acid (HF) solution with acetone. After 10 min, TOP‐Se 0.5 ml (0.5 mmol, 1 M‐TOP solution) was injected into the reaction mixture and maintained for 30 min. The reaction mixture was then cooled to RT. The resulting QDs were precipitated and washed with a 1:1 mixture of acetone and ethanol.

### Materials and Device Fabrication on Glass

Prior to the solution process, the ITO/glass substrates were cleaned by sequential sonication using detergent, deionized water, acetone, and isopropanol for 15 min each time. After sonication, UV‐ozone treatment was per‐formed for 10 min. Then, the ZnO precursor was dis‐solved in 1 ml of 2‐methoxyethanol with 0.1 g of Zn(OAc)_2_ dehydrate and 0.028 mg of ethanolamine with overnight stirring. The prepared solution was spin‐coated onto the cleaned substrates and then heat‐treated at 200°C for 30 min. The prepared QD solution was spin‐coated onto different ZnO substrates. For the QD layer step, the Manganese selenium (Mn_2_Se_2_)‐exchanged QD solution was cast onto the ZnO substrate six times at a spinning rate of 2500 rpm for 30 s. All coating processes were conducted under a dry N_2_ environment in a glove box. After the solution‐fabrication step, MoO_3_ (9 nm) and Au (100 nm) electrodes were sequentially thermally deposited on the substrate through a shadow mask under a vacuum pressure of 2.0 × 10^−6^ Torr.

### Characterization Procedures

Absorption spectra were measured using a SCINCO PDA S‐3100 UV‐vis spectrophotometer. Emission spectra were measured using a JASCO FP‐6500 fluorescence spectrometer. The nanoparticles were dispersed in hexane and spread on a copper grid and silicon wafer for these measurements. TEM images were collected using an FEI Tecnai TITAN 80–300TM transmission electron microscope with an acceleration voltage of 150 kV. High‐resolution TEM images were obtained on an FEI Titan microscope operated at 300 kV. SEM images and X‐ray mapping data were obtained using a JSM‐6700F field emission scanning electron microscope equipped with an INCA energy dispersive X‐ray spectrometer at an operating voltage of 30 kV.

### Electrical Characterization of the QD‐PDs

The *J*−*V* curves were obtained using a semiconductor analyzer (Keithley 4200‐SCS) under dark conditions and 1520 nm light illumination. Spectral R values were obtained from the EQE spectra of the devices, which were measured using an incident photon‐to‐current conversion efficiency system consisting of a Xe lamp (Newport, 100 W) and monochromator. −3 dB Frequency measurements were obtained using an oscilloscope (RIGOL, MSO5354), a preamplifier (SRS, SR570), a function generator (RIGOL, DG1032), and a 980 nm laser (OZ‐2000). The morphologies of the BHJ layers were examined using TEM (JEOL, JEM‐2100F). The UV−vis absorption spectra of the QD films were measured using a UV−vis spectrophotometer (Jasco, V‐770).

## Conflict of Interest

The authors declare no conflict of interest.

## Supporting information

Supporting Information

## Data Availability

The data that support the findings of this study are available on request from the corresponding author. The data are not publicly available due to privacy or ethical restrictions.
