# Supplementary material for: Colloidal InSb Quantum Dots for 1500 nm SWIR Photodetector with Antioxidation of Surface
Source: Adv Sci (Weinh). 2023 Nov 30;11(4):2306439. doi: 10.1002/advs.202306439 (PMC10811490; doi:10.1002/advs.202306439)
Supplement: Supplementary file 1 — Supporting Information [file ADVS-11-2306439-s001.pdf]

ADVANCED SCIENCE
Open Access

Supporting Information

for *Adv. Sci.*, DOI 10.1002/advs.202306439

Colloidal InSb Quantum Dots for 1500 nm SWIR Photodetector with Antioxidation of Surface

Haewoon Seo, Hyeong Ju Eun, Ah Yeong Lee, Hang Ken Lee, Jong H. Kim and Sang-Wook Kim**

Supporting Information

Colloidal InSb quantum dots for 1500 nm SWIR photodetector with antioxidation of surface

Haewoon Seo, Hyeong Ju Eun, Ah-Yeong Lee, Hang Ken Lee, Jong H. Kim and Sang-Wook Kim**

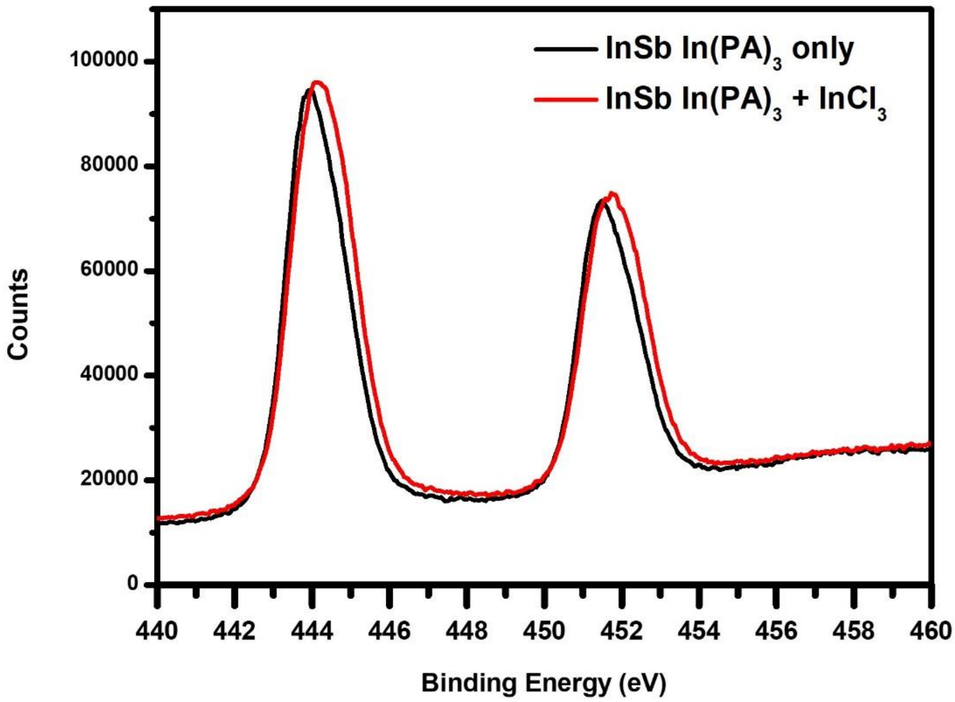

Figure S1. XPS spectra of In 3d in InSb cores synthesized by $In(PA)_3$ only (black line) and the mixture of $InCl_3$ (red line).

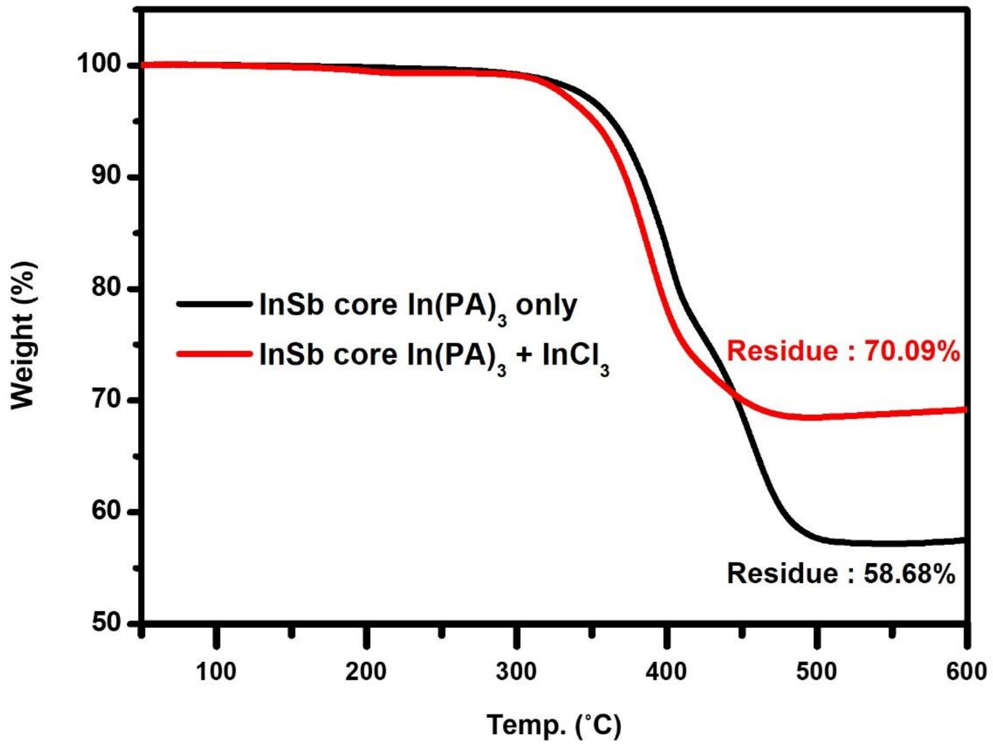

Figure S2. TGA spectra of InSb cores synthesized by $In(PA)_3$ only (black line) and the mixture of $InCl_3$.

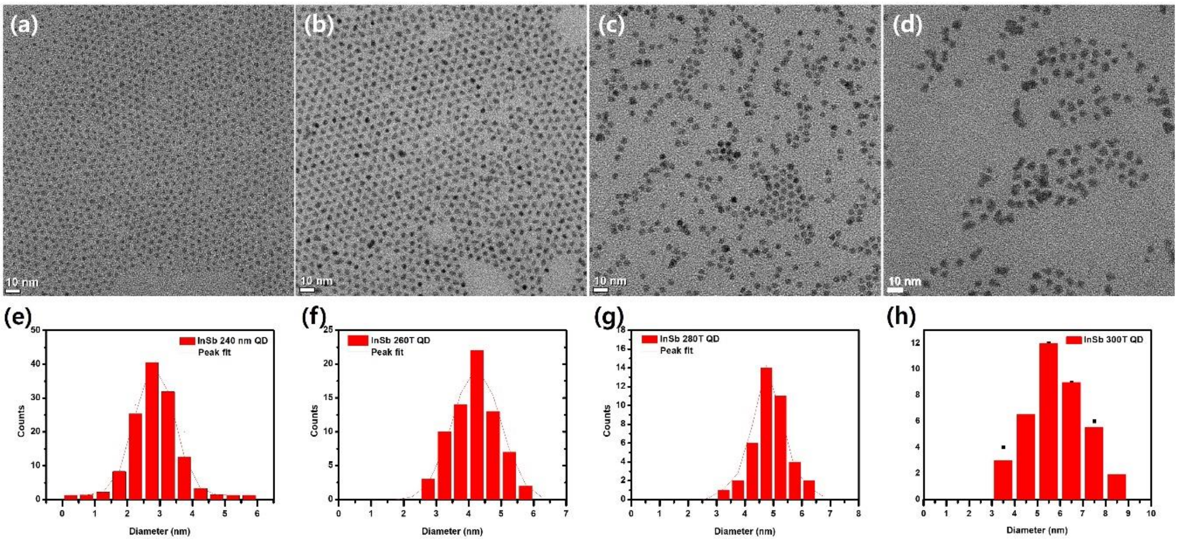

Figure S3. TEM image of InSb QDs depending on growth temperature (a : 240 °C, b : 260 °C, c : 280 °C, d : 300 °C) and calculated the size distribution data (e-h).

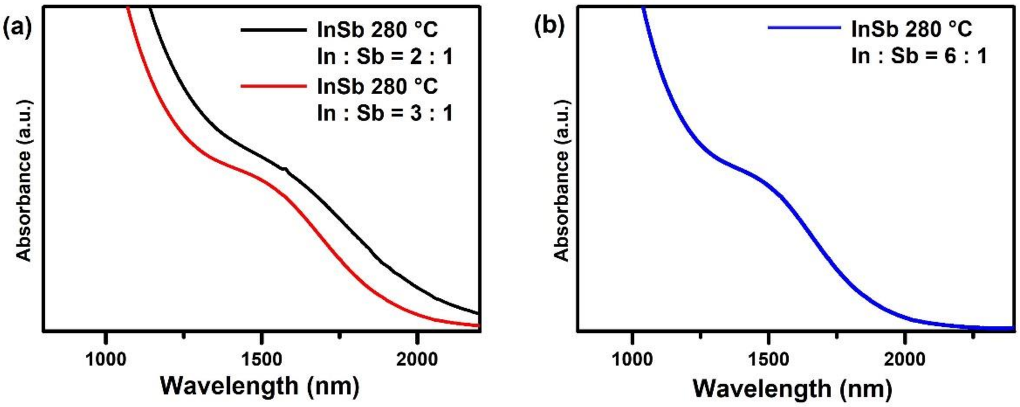

Figure S4. Absorption of InSb 280 °C growth core dependent on In/Sb ratio in synthesis step (a) In : Sb = 2 : 1 (black line) and In : Sb = 3 :1 (red line). (b) In : Sb = 6 : 1)..

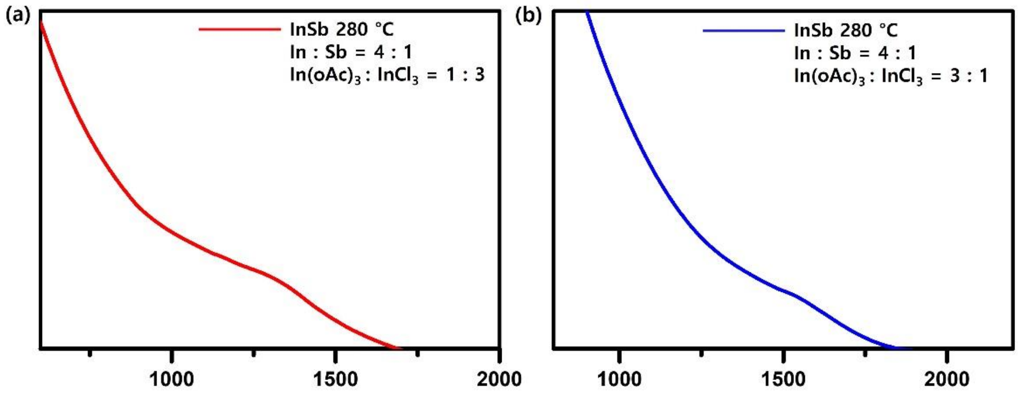

Figure S5. Absorption of InSb 280 °C growth core dependent on $In(PA)_3$ / $InCl_3$ ratio in synthesis step. (a) $In(PA)_3$: $InCl_3$ = 1 : 3 (b) $In(PA)_3$: $InCl_3$ = 3 : 1.

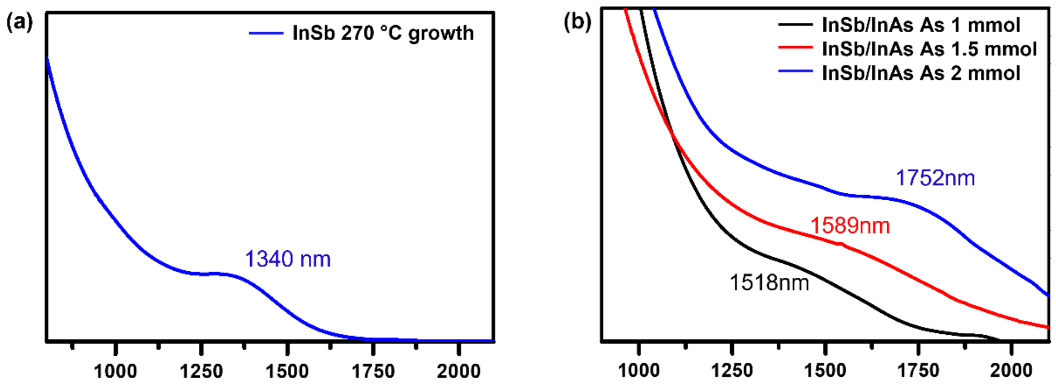

Figure S6. (a) Absorption of InSb 270 °C growth core QDs (b) Absorption of InSb/InAs core/shell QDs dependent on InAs complex amount.

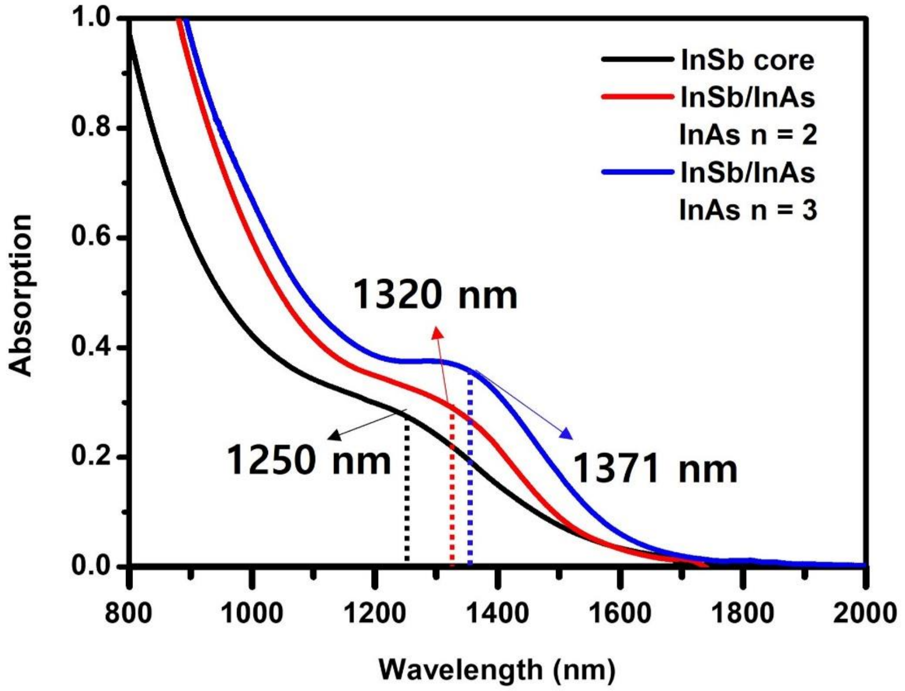

Figure S7. Absorption spectrum of InSb/InAs core/shell QDs dependent on InAs complex amount.

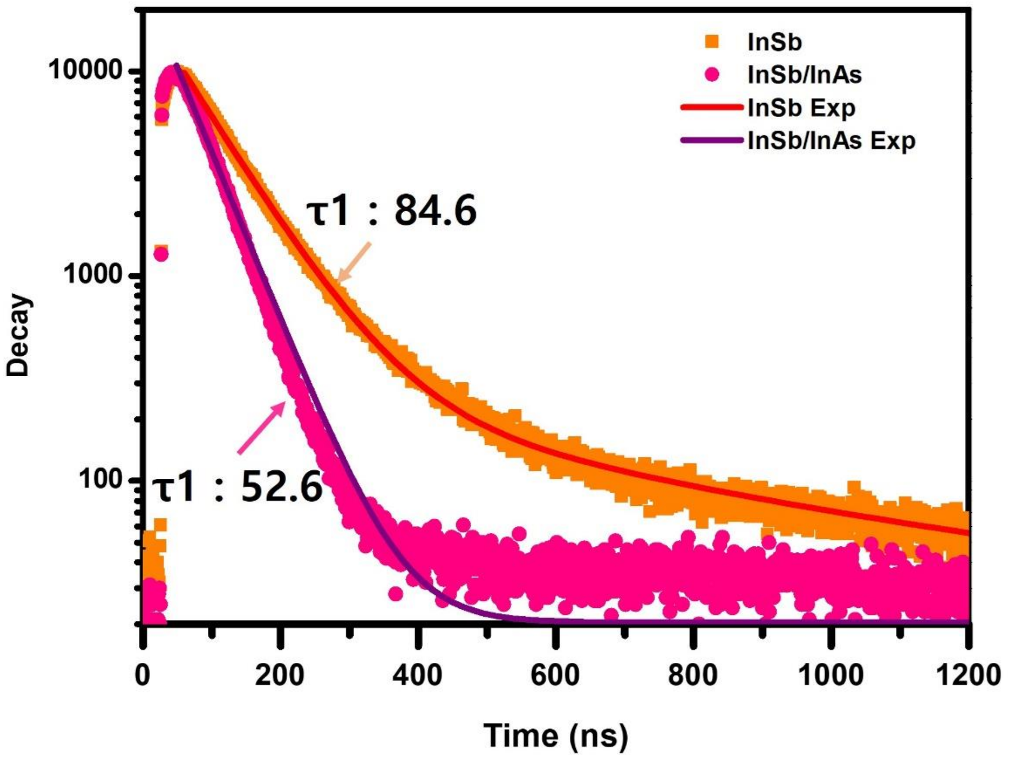

Figure S8. Time-resolved PL decay plot of InSb core and InSb/InAs core/shell QDs.

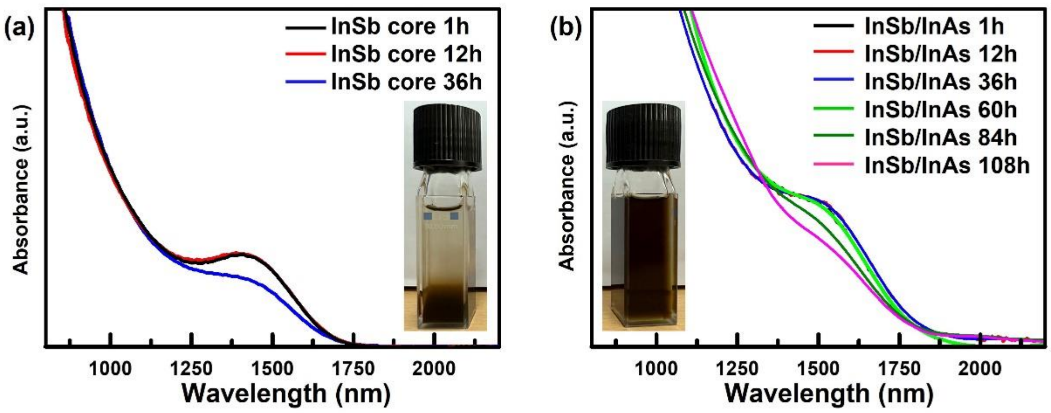

Figure S9. Absorption data change at 120 °C of InSb core and InSb/InAs core/shell QDs in TCE solution. (a) InSb core and (b) InSb/InAs core/shell QDs. (Inset picture : photographs of each QD after 36 hrs)

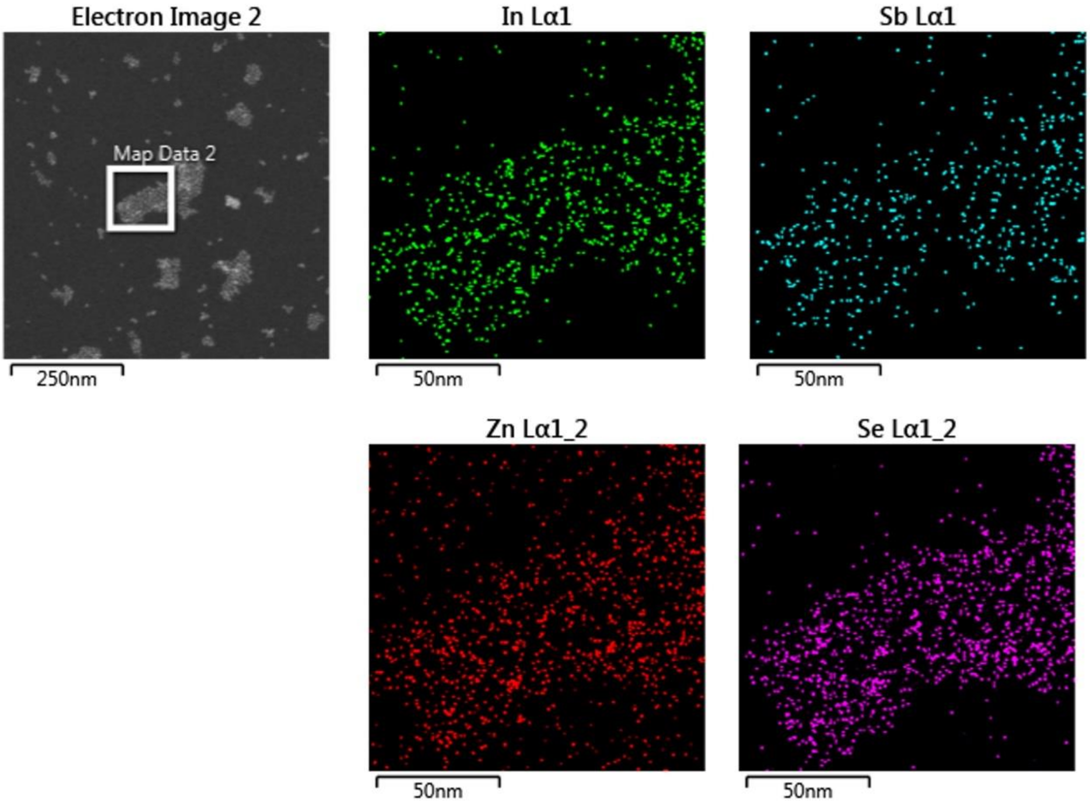

Figure S10. TEM-EDS image of InSb/InAs/ZnSe core/shell/shell QDs.

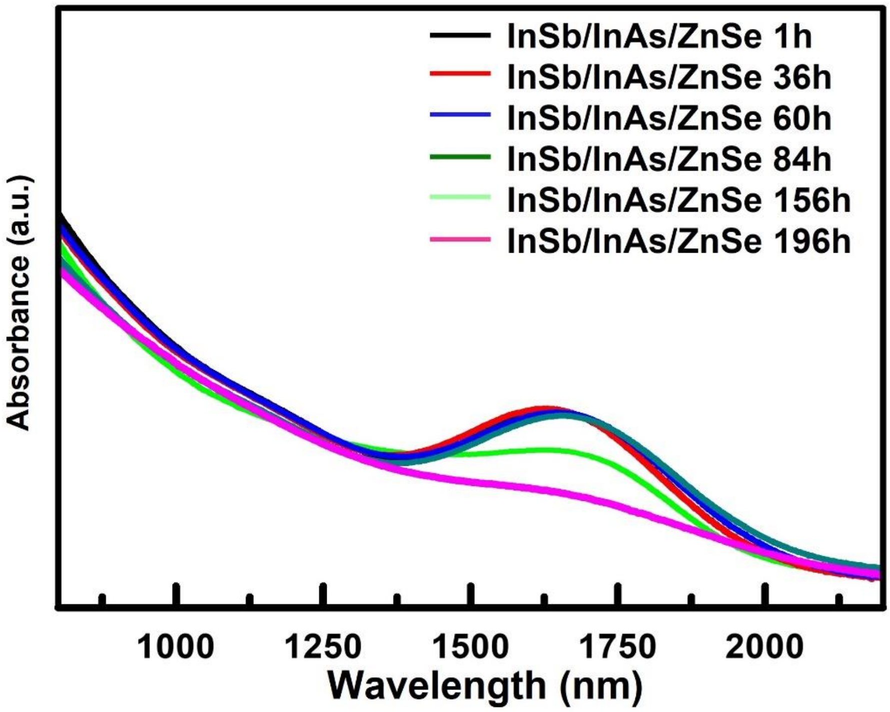

Figure S11. Absorption data change of InSb/InAs/ZnSe core/shell/shell QDs in TCE solution at 120 °C.

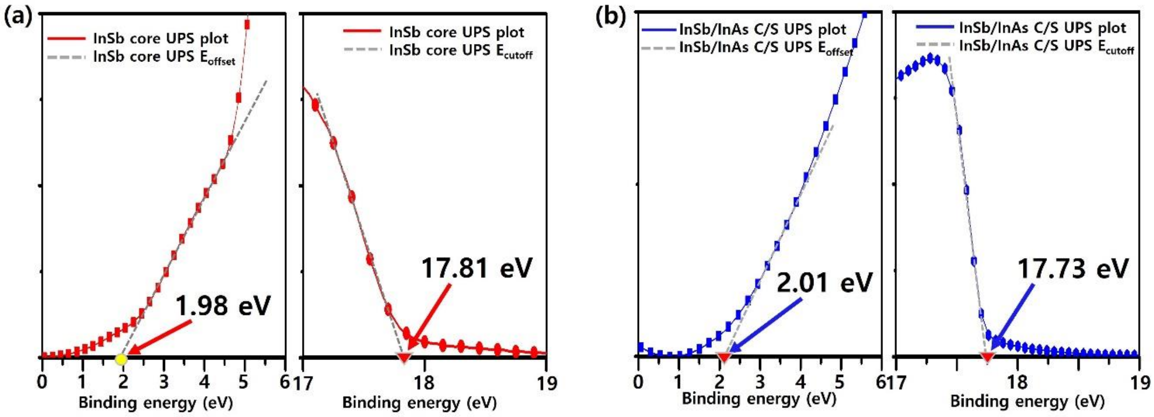

Figure S12. UPS plot of (a) InSb-Mn_2Se_2 and (b) InSb/InAs-Mn_2Se_2 film.

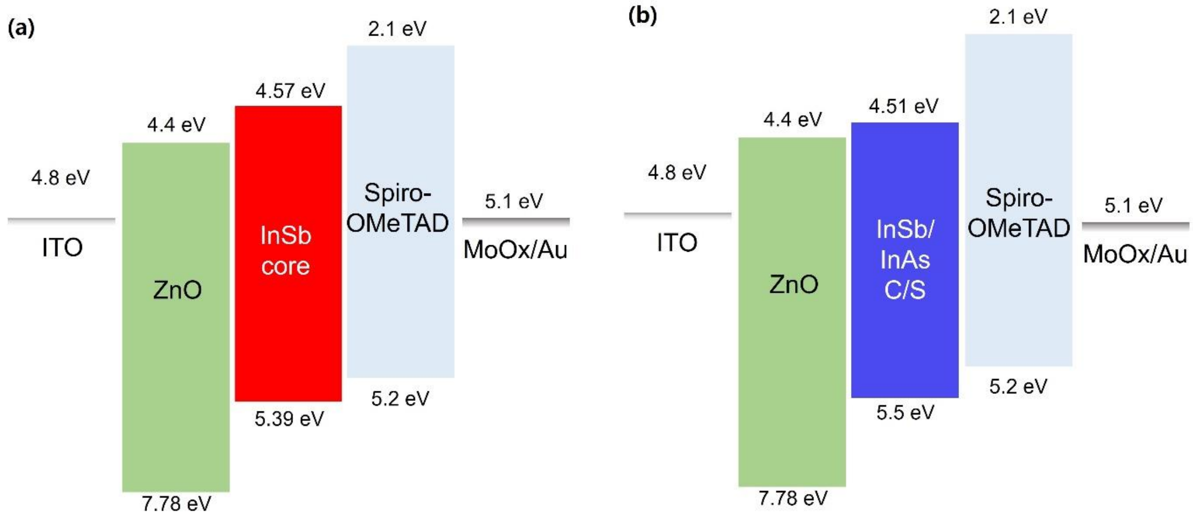

Figure S13. Energy band-diagram of photodetector devices based on (a) InSb-Mn_2Se_2 and (b) InSb/InAs-Mn_2Se_2.

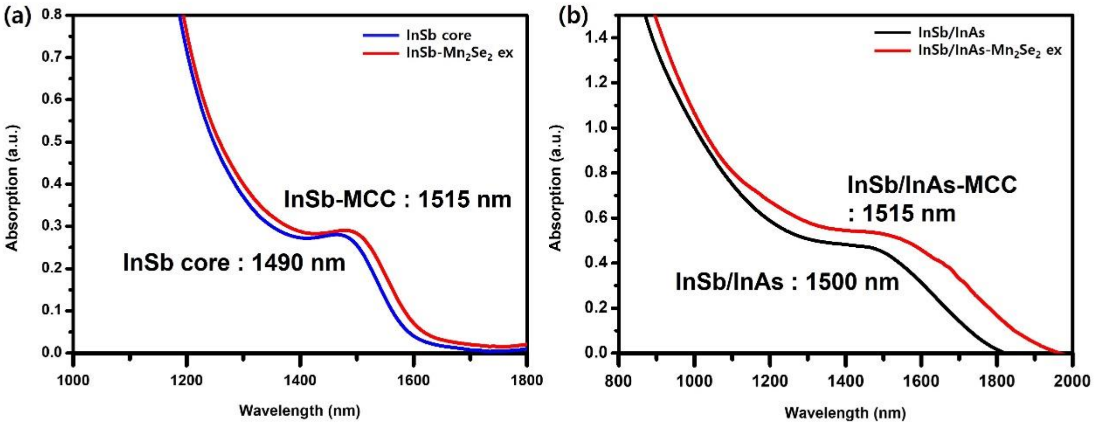

Figure S14. Absorption spectra prior to and after the ligand exchange process. (a) InSb and InSb-Mn_2Se_2 (b) InSb/InAs and InSb/InAs-Mn_2Se_2.

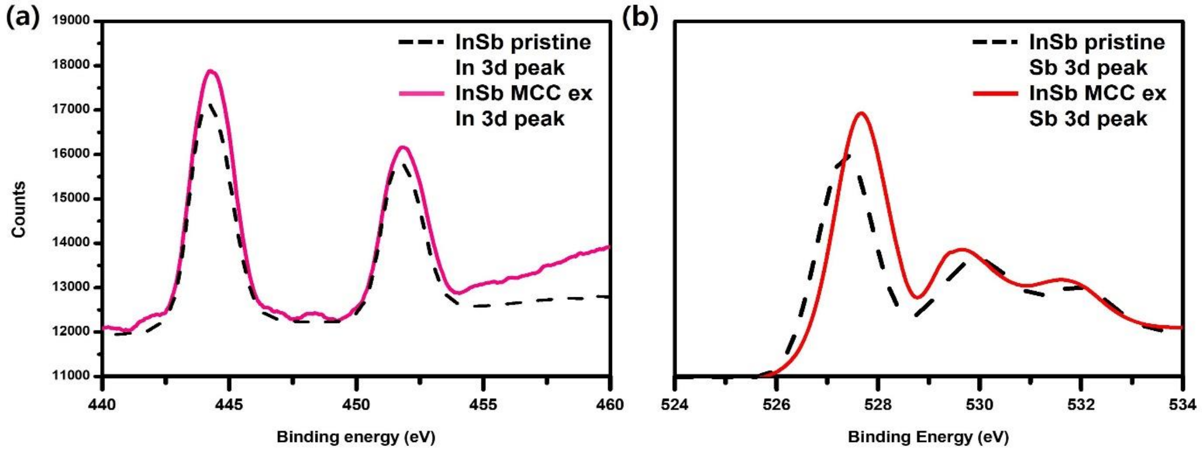

Figure S15. XPS specta of InSb-Mn_2Se_2 film; (a) In_{3d} (b) Sb_{3d}.

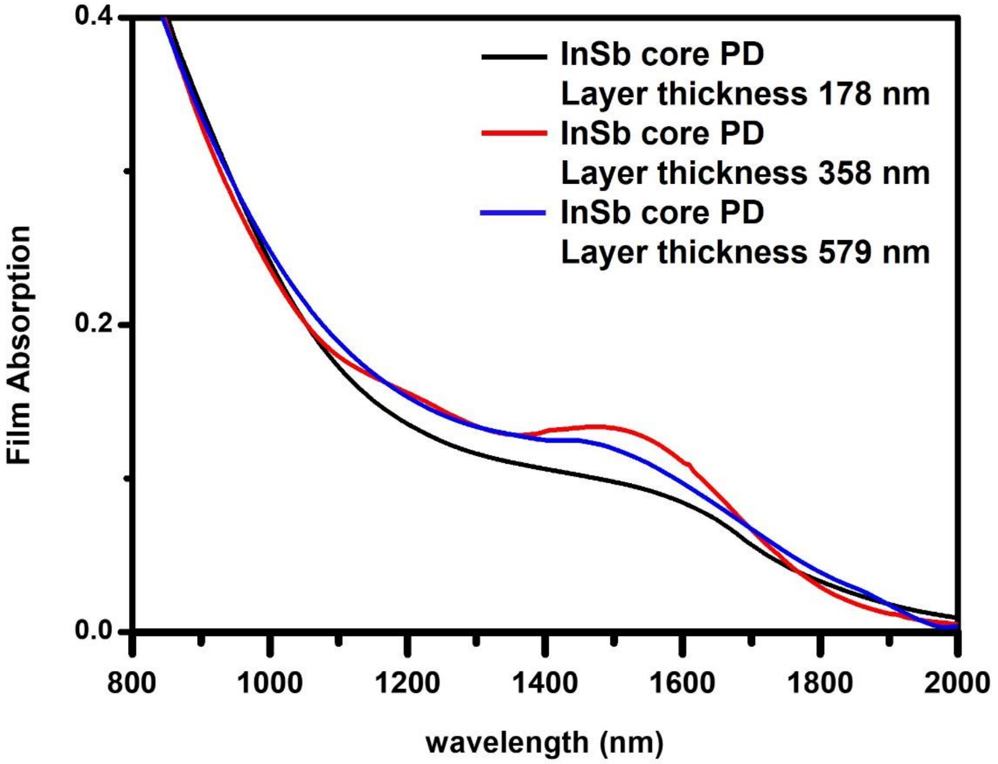

Figure S16. Film absorbance spectra depending on photoactive layer thickness.

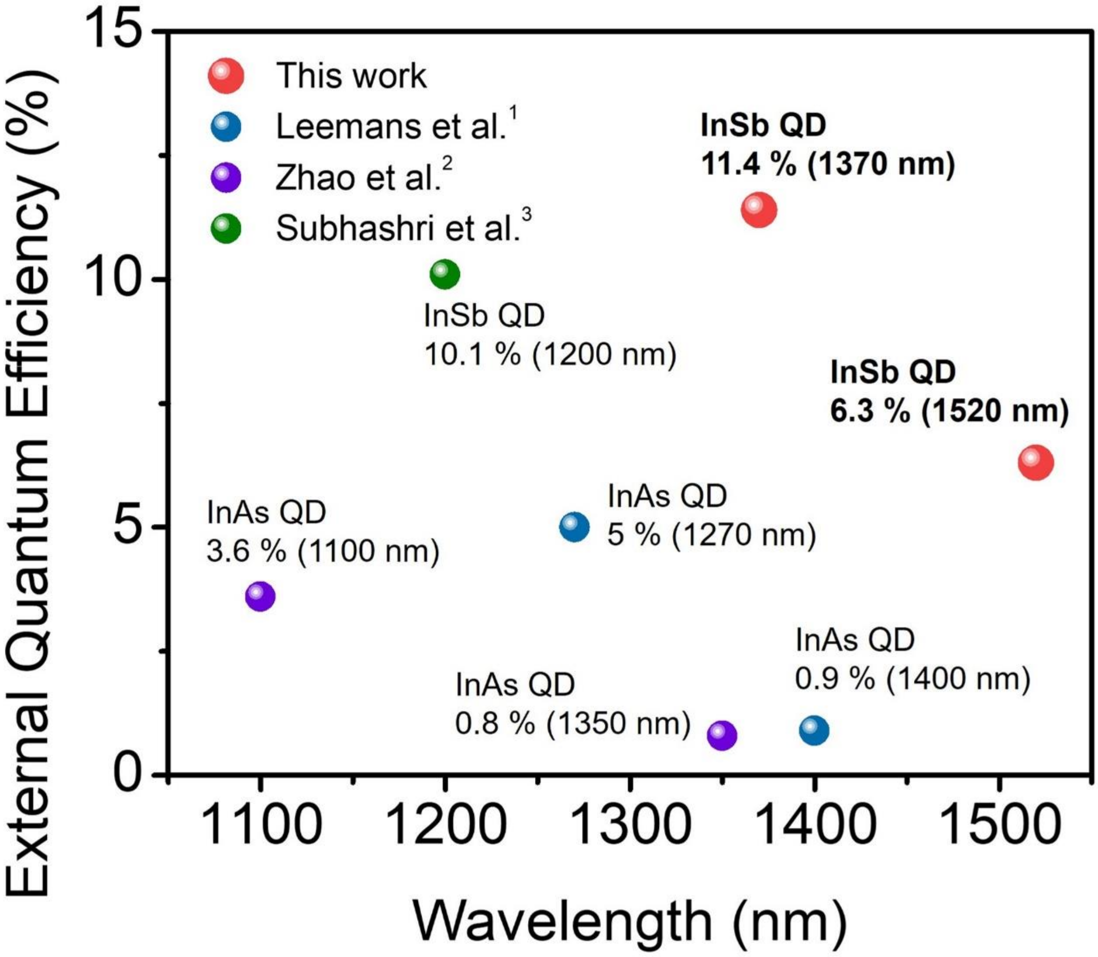

Figure S17. A summary of the comparison of external quantum efficiency (EQE) in quantum dot device performance with respect to previous reports.

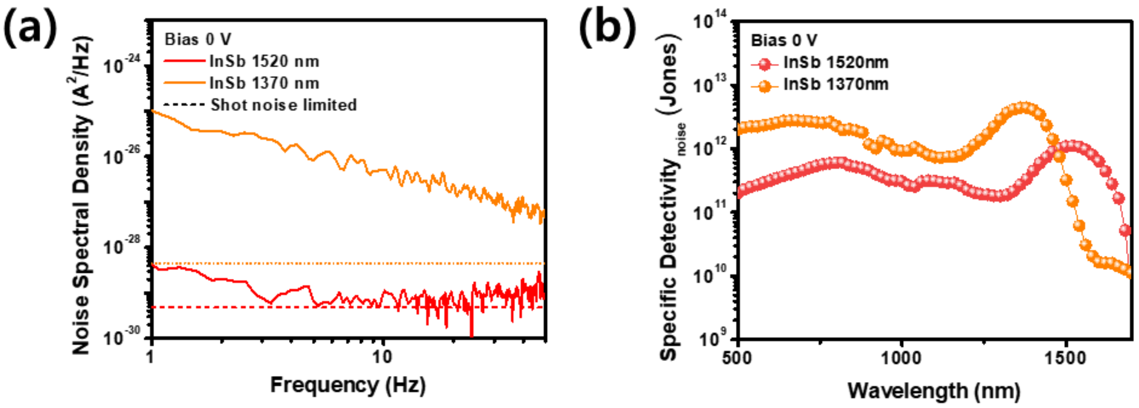

Figure S18. (a) Noise spectral density depending on frequency, and (b) calculated specific detectivity based on the noise spectral density under zero bias (Orange: the 1370 nm device, red: the 1520 nm device).

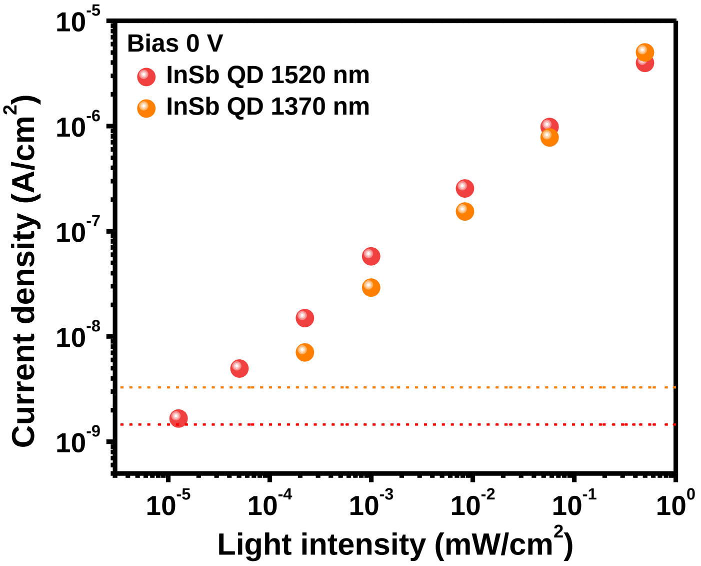

Figure S19. Linear dynamic range of the InSb core devices depending on incident light intensity (the 1370 nm device (orange) irradiates light with a wavelength of 1360 nm, and the 1520 nm device (red) irradiates light with a wavelength of 1520 nm).

Table S1. Comparison table of previous InSb synthesis report.

Ref.	Abs. peak [nm]	Particle [nm]	Core/shell	Device	EQE
Zhao et al.[2]	1200 – 1700	3 – 6.4	X	Phototransistor	3.6% (1100 nm)<br>0.8% (1350 nm)
Liu et al.[3]	1200 – 1750	3.3 – 6.5	InSb/CdS	FET	X
Subhashri et al.[4]	X	4 - 12	X	Photodiode	10.1% (1200 nm)
Crisp et al.[5]	X	2 – 20	X	X	
Tamang et al.[6]	X	4 – 20	X	X	
Zhang et al.[7]	X	54 – 182	X	X	
Bussato et al.[8]	1000 – 1500	2.8 – 6	X	X	
**This work**	**990 – 1700**	**2 – 6**	**InSb/InAs**<br>**InSb/InAs/ZnSe**	**Photodiode**	**11.4% (1370 nm)**<br>**6.3% (1520 nm)**

References

[1] J. Leemans, V. Pejović, E. Georgitzikis, M. Minjauw, A. B. Siddik, Y. -H. Deng, Y. Kuang, G. Roelkens, C. Detavernier, I. Lieberman, P. E. Malinowski, D. Cheyns, Z. Hens, *Adv. Sci.* **2022**, *9*, 2200844.

[2] W. Liu, A. Y. Chang, R. D. Schaller, D. V. Talapin, *J. Am. Chem. Soc*. **2012**, *134*, 20258.

[3] T. Zhao, N. Oh, D. Jishkariani, M. Zhang, H. Wang, N. Li, J. D. Lee, C. Zeng, M. Muduli, H. -J. Choi, D. Su, C. B. Marray, C. R. Kagan, *J. Am. Chem. Soc*. **2019**, *141*, 15145.

[4] S. Chaterjee, K. Nemoto, B. Ghosh, H. -T. Sun, N. Shirahata, *ACS Appl. Nano Mater*. **2023**, *6*, 15540.

[5] R. W. Crisp, G. Grimaldi, L. D. Trizio, W. H. Evers, N. Kirkwood, S. Kinge, L. Manna, L. D. A. Siebbeles, A. J. Houtepen, *Nanoscale* **2018**, *10*, 11110.

[6] S. Tamang, K. Kim, H. Choi, Y. Kim, S. Jeong, *Dalton Trans.* **2015**, *44*, 16923.

[7] K. Zhang, Y. Wang, W. Jin, X. Fang, Y. Wan, Y. Zhang, J. Han, L. Dai, RSC adv. **2016**, *6*, 25123.

[8] S. Busatto, M. de Ruiter, J. T. B. H. Jastrzebski, W. Albrecht, V. Pinchetti, S. Brovelli, S. Bals, M. -E. Moret, C. M. Donega, *ACS nano* **2020**, *14*, 13146.
